# A Systematic Prediction of Multiple Drug-Target Interactions from Chemical, Genomic, and Pharmacological Data

**DOI:** 10.1371/journal.pone.0037608

**Published:** 2012-05-30

**Authors:** Hua Yu, Jianxin Chen, Xue Xu, Yan Li, Huihui Zhao, Yupeng Fang, Xiuxiu Li, Wei Zhou, Wei Wang, Yonghua Wang

**Affiliations:** 1 Bioinformatics Center, College of Life Sciences, Northwest A&F University, Yangling, Shaanxi, China; 2 Beijing University of Chinese Medicine, ChaoYang District, Beijing, China; 3 Department of Chemical Engineering and Materials Science, Dalian University of Technology, Dalian, China; 4 State Key Laboratory of Crop Stress Biology for Arid Areas and College of Plant Protection, Northwest A&F University, Yangling, Shaanxi, China; Koc University, Turkey

## Abstract

*In silico* prediction of drug-target interactions from heterogeneous biological data can advance our system-level search for drug molecules and therapeutic targets, which efforts have not yet reached full fruition. In this work, we report a systematic approach that efficiently integrates the chemical, genomic, and pharmacological information for drug targeting and discovery on a large scale, based on two powerful methods of Random Forest (RF) and Support Vector Machine (SVM). The performance of the derived models was evaluated and verified with internally five-fold cross-validation and four external independent validations. The optimal models show impressive performance of prediction for drug-target interactions, with a concordance of 82.83%, a sensitivity of 81.33%, and a specificity of 93.62%, respectively. The consistence of the performances of the RF and SVM models demonstrates the reliability and robustness of the obtained models. In addition, the validated models were employed to systematically predict known/unknown drugs and targets involving the enzymes, ion channels, GPCRs, and nuclear receptors, which can be further mapped to functional ontologies such as target-disease associations and target-target interaction networks. This approach is expected to help fill the existing gap between chemical genomics and network pharmacology and thus accelerate the drug discovery processes.

## Introduction

As is well known, the identification of novel promising drugs and targets, as a time-consuming and efforts-costing process, is quite a hard goal to achieve. For instance, in 2006 only 22 new molecular entities were approved by the Food and Drug Administration (FDA) despite the astronomical research and development expenditures as high as up to $93 billion USD [1]. One crucial cause for this situation may be the existence of abundant potential drug-target interactions which have not been discovered so far. Although various biological assays are becoming available, experimental qualification of drug-target interactions remains challenging and expensive even nowadays [2], [3]. Actually, it is estimated that the set of all possible small molecules has already consisted of more than 10^60^ compounds [4], which creates incredibly great difficulties in comprehensive understanding of the interface between chemical space and biological systems [5]. Furthermore, plentiful evidences have exhibited that the patterns of drug-target interactions are too various to be understood as simple one-to-one events [6], [7], due to the reasons of (1) structurally different drugs might express similar activities and bind to the same proteins, and (2) one drug might exert impacts on multiple targets. Hence, there is a strong incentive to develop appropriate theoretical computational tools which are capable of detecting the complex drug-target interactions.

Currently, the most widely used methods are the ligand-based virtual screening (LBVS), structured-based virtual screening (SBVS) and the text mining-based approach. Theoretically, LBVS compares candidate ligands with the known drugs of a target protein to find new compounds using statistical tools [8], [9]. However, the performance of LBVS is often poor when the number of known active molecules for a target of interest is too small. Moreover, this method generally has difficulty in identifying drugs with novel structural scaffolds that differ from the reference molecules. Different from LBVS, SBVS is constrained by the available crystallographic structure of target, thus hindering the prescreening process by *in silico* tools. And this problem is particularly serious for those membrane proteins, like the GPCRs (G-protein coupled receptors) whose 3D structure information is still unavailable up to date [10]. The above two methods are predictive approaches that provide with novel, testable small molecule-target associations, while the text mining-based approach is a way to gather information previously existing in the literature that would probably have been missed. Additionally, it also suffers from an inability to detect new biological findings, and their efficiency is generally hampered by the redundancy of the compound and gene names in literature [11]. Therefore, the genome-wide application of LBVS, SBVS and texting mining-based methods still has many limitations.

An effective means that might overcome these problems is not to considerate each drug or target independently from other drugs or targets, but to take the standpoint of chemical genomics [12] which could open up new opportunities to identify new drug leads or therapeutic targets instead. Chemical genomics aims at exploiting the whole chemical space, which corresponds to not only the space of the small molecules but also of those proteins (drug targets) interacting with the molecules [13]. Recently, several chemical genomics approaches, including the ligand-based, target-based or target-ligand methods have been developed to predict the interactions between compounds and proteins [14]–[20]. The ligand-based method that integrated the protein targets was designed at the level of families or subfamilies which is appropriate for some specific protein families such as GPCRs [14], [15]. Based on the ligand binding site similarity, Frimurer et al developed a target-based approach which clustered the receptors and pooled together the known ligands for each cluster to infer shared ligands [16]. Different from these two approaches, the target-ligand approach combines the ligand chemical space, target space and the currently known drug-target networks information to construct a complex forecast system, with purpose to predict ligands or targets for a given target or ligand without prior attempting to define a special set of similar receptors or ligands. For instance, the amino acid sequences, 2-dimensional chemical structures and mass-spectrometry data have been collected together to build a statistical method for predicting compound-protein interactions based on 519 approved drugs and their 291 associated targets [17]. Similarly, the chemical functional groups and biological features have also been adopted to establish the classification models for predicting the drug-target interaction network [18]. Interestingly, without the negative samples, the semi- supervised machine learning algorithm NetLapRLS has been developed based on heterogeneous biological data, which could effectively predict the interaction of each chemical-protein pair [19]. Furthermore, based on DrugBank database, the sets of chemical substructures and protein domains have also been collected and effectively analyzed using Sparse Canonical Correspondence Analysis (SCCA) and Support Vector Machine (SVM) methods to identify molecular recognition rules between drugs and targets [20]. However, all these aforementioned methods might suffer from the small receptor space which only focused on certain protein families or the limited chemical space only covered by the FDA approved drugs.

To predict the drug-target interactions, we have designed a set of *in silico* tools by incorporating the chemical, genomic and pharmacological information into an integrated framework using the largest available dataset of DrugBank database. The predictions are based on extraction of conserved patterns from subdivided interaction vectors involving both proteins and their corresponding ligands (protein and ligand encoding vectors). A notable advantage of these tools is that it allows us to take proteins of different families into accounts thanks to the choice of protein encoding by the structural and physicochemical properties derived from their primary sequences. The powerful ensemble-based method, i.e., Random Forest (RF), was adopted to construct the models, which is more robust against the overfitting problem and performs more efficiently for large-scale data sets when compared with some traditional statistical methods such as Linear Discriminant Analysis (LDA), the Partial Least Square (PLS) and Aritificial Neural Network (ANN) [21]. The performance of the RF algorithm was compared with that of the SVM method to validate the reliability of the obtained models. The validated models were further employed to systematically predict the known/unknown drugs or targets involving the enzymes, ion channels, GPCRs and nuclear receptors, etc. Particularly, we successfully identify unrelated target proteins of chemical compounds using RF method, and meanwhile, effectively distinguish the novel scaffold hopping ligands of the receptors, which will significantly facilitate the drug-target discovery.

## Materials and Methods

### Benchmark Dataset

Dataset for drugs and targets with known pharmacological interactions were extracted from DrugBank database (http://drugbank.ca/, accessed on June 1st 2011), which so far contains 6707 drug entries including 1436 FDA-approved small molecule drugs, 134 FDA-approved biotech (protein/peptide) drugs, 83 nutraceuticals and 5086 experimental drugs. Additionally, 4228 non-redundant protein (i.e. drug target/enzyme/transporter/carrier) sequences are also potentially linked to these entries. To confirm the quality of this data set, we have carefully compared this database with other databases such as STITCH, SuperTarget and KEGG database, as well as the literature [22], [23]. In the process of building dataset, some drugs and targets (such as nitric oxide and ribosomal protein Thx) were omitted since their chemical descriptors cannot be calculated (details are provided in Supporting Information S1). As a result, a dataset including 6511 drugs and 3987 targets was applied in this work as the benchmark dataset (detailed information of these drugs and targets was given in Supporting Information S2 and S3).

### Chemical and Protein Descriptors Calculation

Chemical descriptors were calculated using DRAGON program (http://www.talete.mi.it/index.htm), which was designed to execute the computation of 20 molecular descriptors categories including 1664 descriptors such as constitutional descriptors, topological descriptors, 2D autocorrelations, topological charge indices, eigenvalue-based indices and molecular properties et al. (details are referred to DRAGON manual). In this work, the charge descriptors and some other descriptors such as IC2, TIC2, SIC2 and CIC2 were discarded as these descriptors cannot be calculated for all drugs (Supporting Information S4). Additionally, in this work, some constant descriptors and near constant descriptors were also removed. And finally, 1080 descriptors were used for subsequent analysis. The protein sequence descriptors were calculated using PROFEAT WEBSEVER (http://jing.cz3.nus.edu.sg/cgi-bin/prof/prof.cgi), involving descriptors like Moran autocorrelation, Dipeptide composition and so on [24]. In order to handle varying sequence length and to extract protein features to the largest extent, each protein was represented by a set of structural and physicochemical descriptors derived from their primary sequences including Amino acid composition descriptors; Dipeptide composition descriptors; Autocorrelation descriptors; Composition, Transition, Distribution descriptors; Quasi-sequence-order descriptors; Amphiphilic pseudo-amino acid composition descriptors and Total amino acid properties descriptors, which can transform the changeable length of protein sequence to a standard feature vector of 1080 dimensions. The detailed information of the descriptors was provided in Supporting Information S5.

### Construction of Training and Test Sets

In order to obtain the experimental dataset, we have constructed a set of numerical vectors for the drug-target pairs (both for positive and negative samples) by concatenating chemical descriptors and protein descriptors. The positive samples were constructed by the known interaction relationships that obtained from DrugBank database. As the information about negative samples was unavailable, a production procedure for negative samples was designed as follows: (I) re-coupled all drugs and targets in the benchmark dataset into pairs, (II) removed those drug-protein pairs existed in the positive samples, and keep the remaining pairs which represent the non-interaction space, (III) randomly picked the negative pairs from the non-interaction space until they reached the same number as the positive pairs. As a result, 13597 positive samples and 13597 negative samples were produced as the experimental dataset. Based on this experimental dataset, we have developed four models according to different external validations, i.e., Model I for “general” prediction, Model II for “new-drug vs known-target” prediction, Model III for “new-target vs known-drug” prediction and Model IV for “new-drug vs new-target” prediction. Here, the “general” means the Model I is an universal model, which can be applied for all possible predictions. And the drugs and targets in the training set are called known drugs and known targets, whereas those not included are termed as new drugs and new targets.

In detail, the procedures for producing training and test sets of the four models were performed as follows: For the Model I, the training and test sets were generated by randomly splitting the experimental dataset. For the other three models, the processes of creating the training and test sets involved two steps. Firstly, the experimental dataset was randomly split into two parts: an initial training set and an initial test set. Then, the training and test sets of Model II were obtained by moving the samples including the known drugs/new targets from the initial test set to the initial training set. The training and test sets of Model III were derived by shifting the samples that contain the new drugs/known targets from the initial test set to initial training set. And the training and test sets of Model IV were produced by deleting the samples including the known drugs/known targets from the initial test set.

Finally, we got the training sets I, II, III, and IV of positive samples 10877, 11148, 11237, 6933 and negative samples 10878, 10593, 10605, 9402, respectively. The test sets I, II, III, and IV contained positive samples 2720, 2449, 2360, 446 and negative samples 2719, 3004, 2992, 596, respectively. In this study, all experimental data were separately pre-scaled to the range from −1 to 1. The information of experimental dataset was provided in Supporting Information S6.

### Random Forest

Recently, attention has been concentrated on using “ensemble learning” method to generate classifiers [25]–[28]. As a relatively new ensemble tool, Random Forest algorithm is firstly proposed by Leo Breiman and Adele Cutler [21] which has four predominant advantages in dealing with pattern recognition problem, i.e., 1) it runs efficiently on high dimensional multiclass datasets, 2) it does not overfit when the number of features exceeds the number of samples, 3) it is robust against noise compared to the boosting method, 4) it adopts the Bagging (bootstrap aggregating) method which can maintain the strength of the trees while reducing their correlation and improve the prediction accuracy.

In addition, RF can be developed as a classifier that consists of many decision trees and outputs the class that is the mode of the classes output by individual trees. Let the number of training cases be 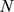, and the number of variables in the classifier be 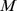, the process of the RF classifier is as follows: (I) The variable 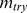 which determines the decision at a node of the tree is defined to be less than 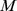. (II) Draw 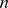 bootstrap samples 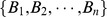 from the original training set. (III) Set up an unpruned tree 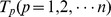 with each training set 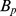. At each node of the tree, randomly choose 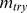 variables on which to make the decision at that node. And then calculate the best splits based on these 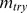 variables. (IV) Predict the class of input samples by the majority votes of the 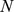 trees.

Two tuning parameters, i.e., the number of trees and 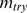, are important in establishing the RF models. Usually, 500 trees are sufficient to generate a model for most cases [21]. 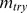 is the number of descriptors randomly sampled as candidates for splitting at each node during the tree induction, ranging from 1 to the total number of the variables (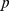). Empirically, the default value of 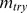 (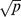 for classification) was assigned to building models, since the performance of RF seems to change very little over a wide range of values except the extreme 1 and 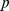 [21], [25]–[28]. Normally, the performance of a pattern recognition model might be severely affected if those irrelevant descriptors are not removed prior to the model training. However, it has been shown that the feature selection is not quite necessary in building the RF models [25]–[28], as the Out-Of-Bag (OOB) error is used to get the estimates of feature importance. In this work, the Random Forest soft package, which was developed by Leo Breiman et al., was used to build the RF prediction models (available at http://www.stat.berkeley.edu/users/breiman/).

### Support Vector Machine

SVM represents a class of statistical learning algorithms that have been widely used in bioinformatics and chemometrics due to its remarkable generalization performance in managing linearly non-separable problems [29]–[31]. Since its theory had been thoroughly described in the literature [32], only a brief description of the method is given here. Given 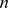 samples, each of which has an 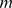-dimensional feature vector 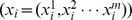 and the two classes 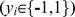 respectively representing the interaction and non-interaction, the classifier is produced as follows:
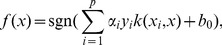
(1)where 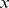 is the new object to be classified, 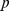 is the number of the training samples, 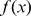 is a decision function and 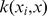 is a kernel function that shows similarity between two vectors. The parameters 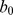 and 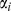 are obtained by solving a quadratic programming problem. For linearly separable cases, SVM constructs a maximal margin hyper-plane to separate the positive samples (interactive pairs of compound-protein) from the negative ones (non-interactive pairs of compound- protein). A new pair of compound-protein can be classified as a positive or negative when 
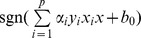
 is positive or negative, respectively. For not linearly separable data, SVM maps the input numerical vectors into a higher dimensional feature space to construct a maximal margin hyper-plane that separate the positive from the negative samples by using a kernel function. And the interactions between the compounds and proteins can be classified as a positive or negative when 
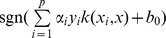
 is positive or negative, respectively. In this study, we have used a portion of the codes from the LIBSVM suite of programs (http://www.csie.ntu.edu.tw/~cjlin/libsvm), which employs a modified version of the sequential minimal optimization (SMO) algorithm [33]. The soft margin SVM was employed to construct the statistical models. Generally, four kinds of kernel functions, i.e. linear function, polynomial function, sigmoid function and radial basis function (RBF), are available to perform prediction. Empirical studies have demonstrated that the RBF outperforms the other three kinds of kernel functions [34]. Hence, this work adopted the RBF to perform inference process. The regularization parameter 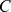 and the kernel parameter 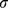 were selected based on the overall accuracy of the internal five-fold cross-validation using the grid search method. The simulation process was developed by using PYTHON (version 3.2) and GNUPLOT (version 4.4).

### Model Validation

In order to fully assess the suitability of these *in silico* models, both the internal and external validations methods were employed. Firstly, all developed models were evaluated by internal five-fold cross-validation. During this process, the training set was randomly divided into five subsets of approximately equal size, where four subsets were selected as the training set to develop a model and the remaining samples as test set for the model validation. The process was repeated five times so that every subset was used as the testing set once. Secondly, we carried out four external independent validations for all models using different test sets. Finally, the performance of the RF models was compared with that of the corresponding models built by SVM method.

### Measurement of Prediction Quality

**Figure 1:**
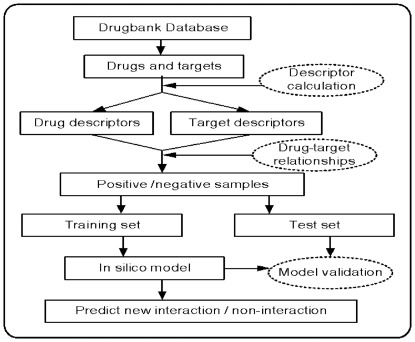
The flowchart of the modeling procedure.

In the case of classification, the assessment of the prediction quality of statistical models is typically performed on the basis of several parameters [35]. The sensitivity (SE) of the present models describes the ratio of correctly predicted interactions to the total number of the drug-target interactions, whereas the specificity (SP) refers to the ratio of correctly predicted non-interactions to the total number of the drug-target non-interactions. The integrated parameter concordance (the ratio of correctly predicted compound-protein pairs to the total number of tested compound-protein pairs, CO) gives an overall model performance value.
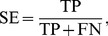
(2)

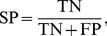
(3)

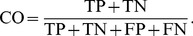
(4)Here, TP and FP are the quantity of the true and false interactions between the drugs and targets, respectively, TN and FN are the quantity of true and false non-interactions between the drugs and targets, respectively. In addition, the performance was also evaluated by using a receiver operating curve (ROC), which is obtained by varying the threshold separating positives from negatives and plotting the TP rate (sensitivity) versus the FP rate (1-specificity). The binding score of RF model in this work was defined as the number of tree vote for 1 (interaction) divided by the number of tree vote for −1 (non-interaction). For all of these statistics, a larger number indicates a better performance of the model. Figure 1 depicts the flowchart of the modeling procedure.

## Results

Recently, various *in silico* methods have been developed to analyze multiple compound-protein interactions. QSAR (quantitative structure-activity relationship) is the most commonly adopted. However, QSAR requires the knowledge of sufficient enough ligands of a given receptor with respect to the complexity of the ligand/non-ligand separation to produce accurate predictors. If few or no ligands are available for a receptor, molecular docking is an alternative approach, which in turn requires the 3D structure of the target. To overcome the shortcomings of these two approaches, several statistical models have been developed to predict the interactions, such as the binary classification models [36], [37] and the supervised bipartite graph inference ones [38], [39]. Although these methods have effectively accomplished the potential drug-target interaction prediction, their applicability domain may still be limited by the small chemical and biological space.

For the purpose of broadening the scope of application of these predictors, we developed a set of *in silico* models based on the large-scale heterogeneous biological data. Our models concatenate the chemical structural and physicochemical properties with the protein structural and physicochemical properties to discriminate the binding patterns from the non-binding patterns. Generally, it is difficult to assess the performance of a chemical and protein feature encoding method in a direct manner. However, if the encoding are biologically meaningful and enable to capture relevant information with respect to receptor-ligand recognition, one would expect that they present good generalization properties. This can be evaluated by using the internal five-fold cross-validation and external independent validation scheme as described in the Materials and Methods section. In the following section, we firstly assess the performance of these obtained models based on these two methods, and then carry out the systematical drug-target interaction predictions to further verify the usefulness of the models in comprehensive prediction.

### Model Evaluation and Comparison

**Figure 2:**
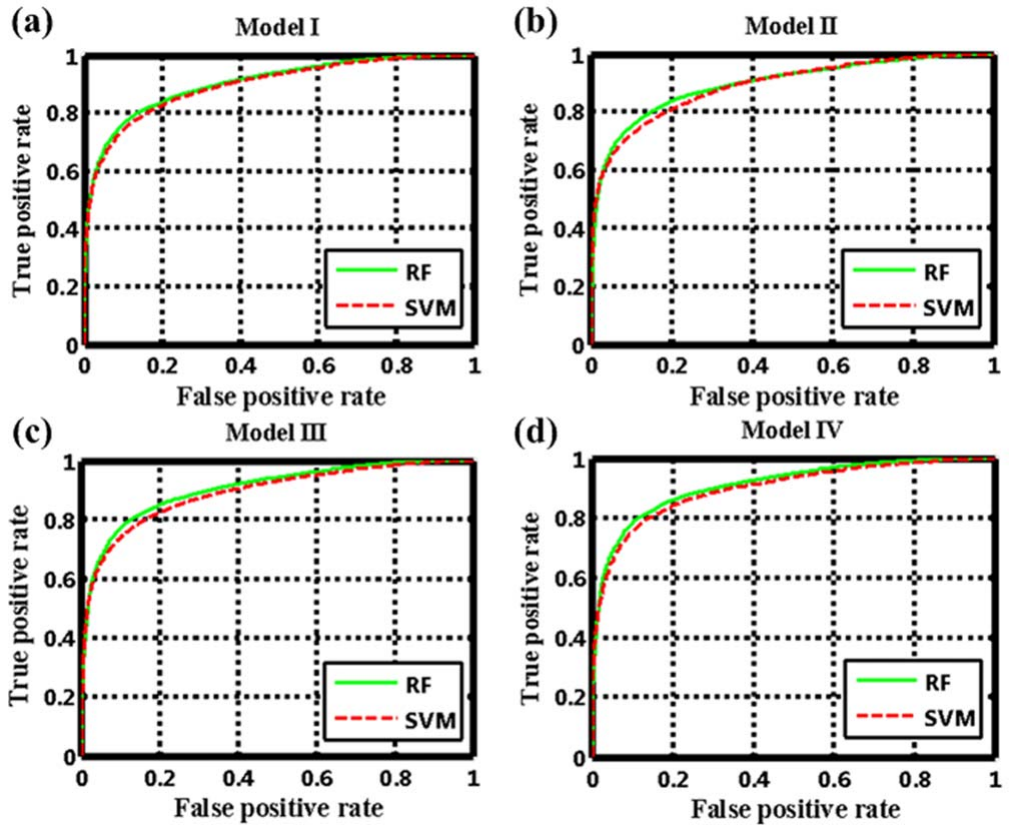
The ROC curves of the RF and SVM in internal five-fold cross validation for (a) Model I, (b) Model II, (c) Model III and (d) Model IV.

To construct the four classification models using the Random Forest method, we firstly validated the predictive performance of these models by the internal five-fold cross-validations. Table 1 shows that these RF models for the internal validation perform consistently well in predicting the binding (average SE 78.50%) and the non-binding (average SP 85.37%) patterns, with an average concordance of 82.26% and an average AUC of 89.65, indicating the strong robustness and capability of the models for prediction of the interactions between drugs and targets. Additionally, the obtained ROC curve (Figure 2) suggests that the RF method can catch sufficient information to detect the drug-target interactions at high true-positive rates against low false-positive rates at any threshold. For example, in the RF Model I, when the true positive rate reaches 40% the false positive rate is as low as ∼2%, and when the true positive rate is 60% the false positive rate is still low as ∼4%.

**Table 1:** Statistics of the prediction performances.

Model	SE (RF/SVM)		SP (RF/SVM)		CO (RF/SVM)		AUC (RF/SVM)	
	Training	Test	Training	Test	Training	Test	Training	Test
Model I	79.58%/76.31%	80.99%/77.50%	84.11%/85.85%	84.66%/85.91%	81.84%/81.08%	82.83%/81.71%	89.50/88.97	90.55/89.91
Model II	81.18%/77.66%	73.66%/74.11%	83.89%/86.11%	83.52%/85.49%	82.50%/81.78%	79.09%/80.38%	90.31/89.79	86.50/87.19
Model III	81.33%/77.56%	52.20%/55.17%	83.22%/85.35%	92.15%/93.62%	82.25%/81.34%	74.53%/76.66%	89.81/89.04	82.77/84.90
Model IV	71.90%/66.41%	32.52%/36.48%	90.25%/88.07%	91.21%/92.42%	82.46%/78.88%	66.09%/68.48%	88.99/85.62	72.64/75.47
Average	78.50%/74.49%	59.84%/60.82%	85.37%/86.35%	87.89%/89.36%	82.26%/80.77%	75.64%/76.81%	89.65/88.36	83.13/83.93

**The AUC (ROC score) is the area under the ROC curve, normalized to 100 for a perfect inference and 50 for a random inference.**

**Figure 3:**
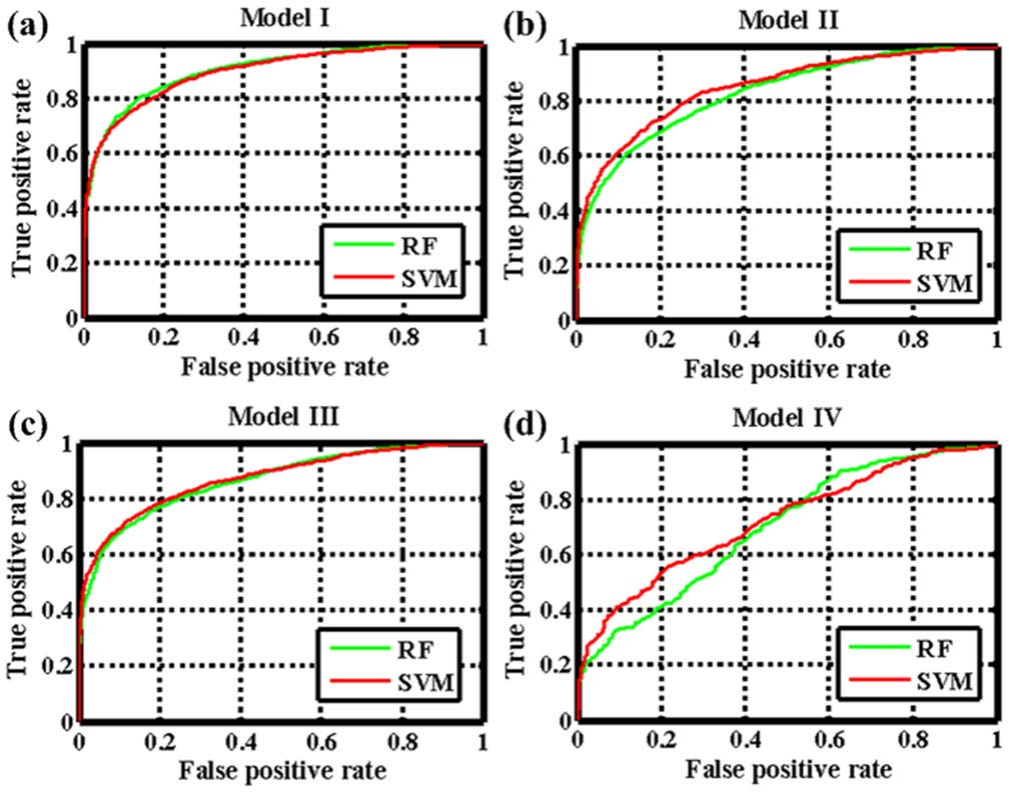
The ROC curves of the RF and SVM in four independent external validations for (a) Model I, (b) Model II, (c) Model III and (d) Model IV.

Subsequently, we used four external independent validations to further assess the generalization ability of these models, i.e., the “general” prediction, the “new-drug vs known-target” prediction, “new-target vs known-drug” prediction and “new-drug vs new-target” prediction (Table 1). For the test set I, the RF Model I acquires a sensitivity of 80.99%, a specificity of 84.66% and a concordance of 82.83%, which are comparable to the results obtained for the internal validation. This suggests that the risk of over-fitting problem is quite low for the obtained models. For the test set II and test set III, RF Model II and III obtain a sensitivity of 73.66% and 52.20%, a specificity of 83.52% and 92.15% and a concordance of 79.09% and 74.53%, respectively. Evidently, the Model II exhibits much better results than model III, and such superiority of Model II is also maintained in the SVM method with the sensitivity of 74.11% and 55.17% for set II and set III, respectively. The reason might be due to: (1) the test set II blends better with the training set II than the test set III with the training set III; (2) the choice of receptor descriptors in this study have more relevant than that of ligand descriptors; and (3) the smaller information space of the receptor protein compared with the ligand chemical in the training set leads to the lack of the available targets information for the test dataset. The predictability for test set IV is relatively weak, with a sensitivity of 32.52%, a specificity of 91.21% and a concordance of 66.09%, respectively. Compared with the previous study, the model built based on this set is not only not improved but also is quite poor in the performance, which may be closely related to the universality and the sample size of test set IV that is not sufficient enough to represent the general cases. The ROC curves of the four external validations are shown in Figure 3, revealing that the prediction capability of the models built on different sets follows an order of Set I>Set II>Set III>Set IV, with a range of the AUC values from 72.64 to 90.55. The above results suggest that the RF learning methods can perform better when a lot of drugs and targets interaction information is available in the learning dataset. Additionally, it also suggests that the adopted encoding method is biologically relevant and enable to efficiently capture the information involved in receptor-ligand interactions.

Based on the same training and test sets as RF, SVM is also applied to build the models. For all the internal validation sets and external set I, the SVM model is slightly worse in both the concordance and the sensitivity than the RF ones. For the external sets II, III and IV, the SVM model is slightly better in the sensitivity, specificity and concordance. The consistency in model performance of the two methods further indicates that these models are robust and reliable for predicting the multiple drug-target interactions. Based on this, we conclude that the conserved binding patterns that are common to the protein families such as GPCRs, nuclear receptors, ion channels and enzymes, can be effectively detected by our proposed approach. It is worth noting that all these models can definitely identify the negative samples (non-interaction) with a quite high specificity from 83.22% to 93.62% for all datasets although the negative samples are initially randomly produced. This from a statistical point of view demonstrates that the drug-receptor recognition is quite specific, thus to find a new drug by chance should be extremely difficult.

**Figure 4:**
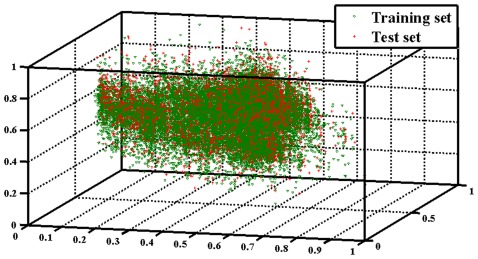
The distribution of all samples of model I using the first three principal components.

In principle, the applicability domain of a classification model is calculated on the basis of the range of individual samples in the training set that the minimum and maximum values of each feature were obtained by considering all the samples of the set. In this work, in order to reduce the dimensionality of the descriptor pool, to eliminate the correlations among variables as well as to retain the information restored in the dataset as much as possible, the principle component analysis (PCA) [40] is applied to the current datasets for analyzing the applicability domain of the obtained models. The distribution of all samples of Model I using the first three PCs is shown in Figure 4 (see others in Supporting Information S7, S8, S9). It can be seen that training and test sets were well distributed in “chemical-biological” space. These results suggest that the applicability domain of these models covers a large part of the whole “chemical-biological” space.

**Figure 5:**
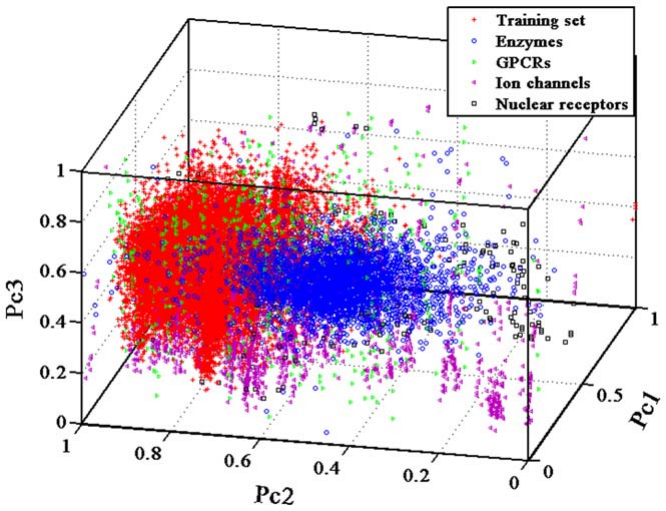
The distribution of the training dataset for Model I and blind testing dataset (including enzymes, GPCRs, ion channel and nuclear receptors) using the first three principal components.

**Figure 6:**
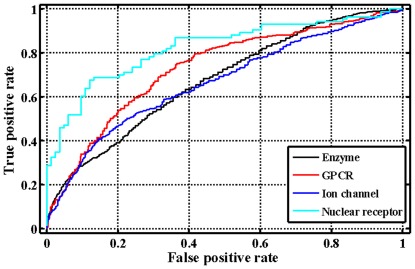
The ROC curves of blind independent validation results for four target protein families.

After confirming the usefulness of our method using the internal five-fold cross-validations and the four external independent validations, we have also conducted a blind testing using an independent dataset obtained from the KEGG database (http://www.genome.jp/kegg/) apart from DrugBank database. In total, 491 compounds and 979 proteins (including enzymes (n = 654), ion channels (n = 204), GPCRs (n = 95) and nuclear receptors (n = 26)) were extracted from this database (Supporting Information S10). The RF Model I for the four datasets obtains an average sensitivity of 47.51%, an average specificity of 74.93% and an average concordance of 61.64% (Table 2). The predicted results for the GPCR and nuclear receptor datasets are fairly good with the sensitivity up to 80.31% and 91.57%, respectively. However, we also observed that the RF Model I might not be capable of predicting the enzyme and ion channel datasets, the obtained sensitivity values are less than 55%. The projection of the first three principal components (Figure 5) shows that most samples of these two datasets deviate from the training dataset of the developed RF model I, which can effectively explain the obtained results. Additionally, the ROC curves in Figure 6 indicate that our methods perform much better than a random inference for the four target protein families, with a range of the AUC value from 66.58 to 82.29. As compared with the recent bipartite graph learning (BGL) model [38] (Table 2), the sensitivity of our proposed approach are much better except the enzyme dataset. It should be also pointed out that our method has the lower specificity and concordance, which might indicate that the negative samples randomly produced from the blind testing dataset contain the potential interaction relationships. All these further suggest that the learning algorithms based on the general chemicals and proteins properties that are related to drug-target interactions, and therefore allow our approach to successfully make predictions. However, we have to confess that there are still a large space to improve the model performances with the development of new descriptors for chemicals and proteins, and even mathematical methods.

**Table 2:** The predicted results for the blind testing sets by the RF model 1.

Dataset	SE (RF/BGL)	SP(RF/BGL)	CO(RF/BGL)	AUC(RF/BGL)
Enzyme	35.82%/57.40%	82.70%/99.50%	59.26%/––	67.43/90.40
GPCR	80.31%/23.40%	55.64%/99.90%	67.98%/––	72.95/89.90
Ion channel	54.09%/27.10%	73.38%/99.60%	63.73%/––	66.58/85.10
Nuclear receptor	91.57%/14.80%	39.76%/99.90%	65.66%/––	82.29/84.30
Average	47.51%/––	74.93%/––	61.64%/––	66.68/––

In addition, using the training set of the Model I, we analyzed the computational cost of the RF and SVM methods for the internal five-fold cross-validation process, which was implemented on a Dell computer (Redhat Linux Operating System) with 2.8 GHz AMD Phenom (tm) II X6 1055T processer and 12 GB RAM. Due to the complexity of the “learning” phase scales with the square of the “number of training compounds times the number of training proteins”, the computation time of SVM (10.86 hours) is much slower than that of the RF (4.53 hours) approach [20]. This indicates that our proposed RF approach works better in terms of computational efficiency.

### Comprehensive Prediction for Potential Drug-Target Interactions

The prediction of drug-target interactions can be directly applied to completing the genome annotations, investigating the drug specificity and promiscuity, and finding the targets for diseases. However, the overall pattern of the interaction interface between the chemical space and biological systems is too large and complex to be captured. For simplicity, the enzyme which has been extensively studied as a class of important drug target family was selected here to illustrate our models' applications of predicting the potential drug-target interactions. A total of 175 enzymes in the DrugBank database were matched with each of 6511 drugs to conduct the comprehensive drug-target interaction prediction using the general RF Model I.

The results show that, in general, two compounds sharing high structural similarity tend to interact with similar target proteins. Likewise, two proteins with high sequence similarity tend to interact with similar drugs. For instance, two structurally similar drugs Adenosine-5′-Diphosphate (DB03431) and Guanosine-5′-Diphosphate (DB4315) were predicted to act on 166 common targets. This indicates a strong interrelation between the receptor partners with their binding counterpart. In addition, we also find that the places for the top scoring drug-target interactions are mainly occupied by several categories of enzymes and drugs, where the enzymes are usually highly homologous such as Prostaglandin G/H synthase 1 and Prostaglandin G/H synthase 2 and the drugs share certain common substructures such as Nicotinamide-Adenine-Dinucleotide (DB01907) and Flavin-Adenine Dinucleotide (DB03147). Interestingly, this demonstrates that only a few families of enzymes and drugs account for the top scoring interactions, which is completely supported by a previous model established based on the bipartite graph learning algorithm [38]. Meanwhile, the results also demonstrate that our RF approach offers better predictions for proteins/chemicals that have been extensively studied and for which many ligands/receptors are known. These results further imply that our proposed methods are able to learn, i.e., the more information is provided, the better the prediction.

Here, we take the top 10 scoring novel interactions as examples to illustrate the above findings. As shown in Table 3, compound Bromfenac (DB00963) is predicted to act on Prostaglandin G/H synthase 1 with a score of 0.992. Actually, this interaction has been confirmed and was annotated in DrugBank database [41]. For Asparagine synthetase [glutamine-hydrolyzing], a new ligand Indomethacin (DB00328) is predicted to bind to it with the score of 0.984, which might be hinted by an indirect experiment in which the Asparagine synthetase expression level was indeed upregulated by this compound [42]. Additionally, Oxyphenbutazone (DB03585), as a well-known nonsteroidal anti-inflammatory agent, binds to the cyclooxygenase (COX) Prostaglandin G/H synthase 1 and Prostaglandin G/H synthase 2 with the same binding scores of 0.982. This is supported by the fact that the nonsteroidal anti-inflammatory drugs could produce therapeutic activities through the inhibition of cyclooxygenase [43]. With no exception, for all the remaining interactions, the predicted ligands of certain receptors are invariably similar in structure with those confirmed ligands as mentioned above. All these outcomes enhance the strength of our proposed methods for realistic drug-target interaction prediction application.

**Table 3:** The predicted top 10 scoring novel drug-target interactions.

Protein name (UniProt ID)	Drug generic name (DrugBank ID)	Binding score
NAD(P)H dehydrogenase [quinone] 1 (P15559)	Flavin-N7 protonated-adenine dinucleotide (DB02332)	0.996
NAD(P)H dehydrogenase [quinone] 1 (P15559)	NADH (DB00157)	0.994
Alcohol dehydrogenase [NADP+] (P14550)	NADH (DB00157)	0.992
Prostaglandin G/H synthase 1 (P23219)	Bromfenac (DB00963)	0.992
Prostaglandin G/H synthase 1 (P23219)	D-allopyranose (DB03989)	0.990
Cholinesterase (P06276)	Beta-D-Glucose (DB02379)	0.988
Cholinesterase (P06276)	D-Allopyranose (DB03989)	0.986
Asparagine synthetase [glutamine-hydrolyzing] (P08243)	Indomethacin (DB00328)	0.984
Prostaglandin G/H synthase 1 (P23219)	Oxyphenbutazone (DB03585)	0.982
Prostaglandin G/H synthase 2 (P35354)	Oxyphenbutazone (DB03585)	0.982

**Figure 7:**
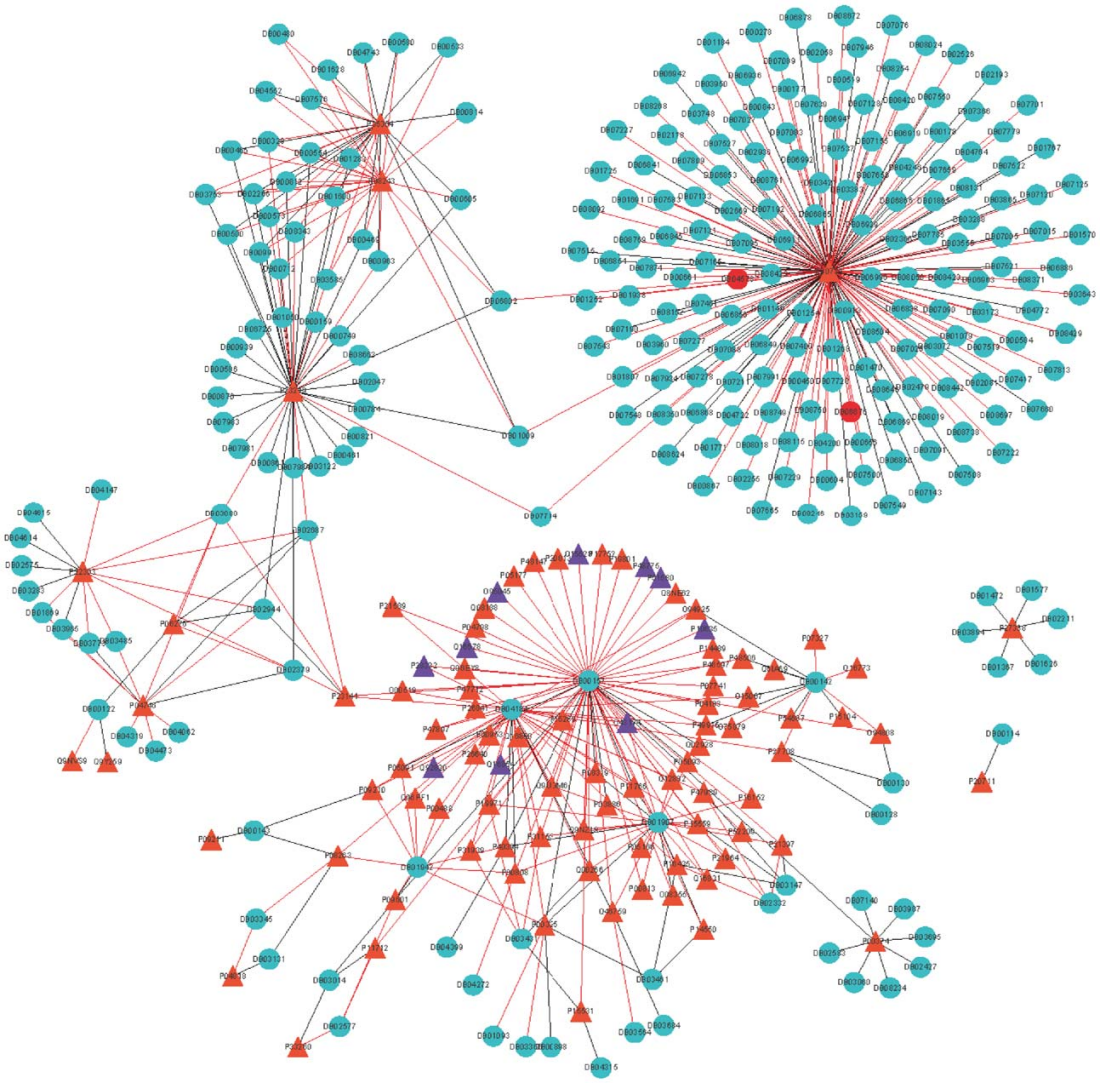
Predicted drug-enzyme interactions with the 500 highest scores, where the triangle and circle nodes indicate the enzymes and drugs, respectively; the orange and purple triangle indicate the known targets and new predicted targets, respectively; the green and red circle indicate the known drugs and new predicted drugs, respectively; the gray and red edges indicate the known interactions and newly predicted interactions, respectively.

**Figure 8:**
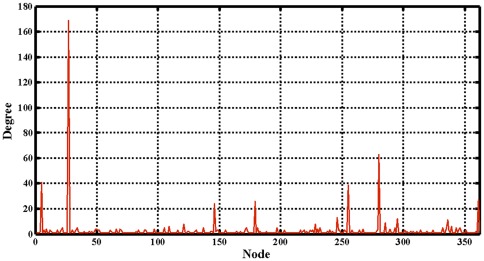
The node degree distribution of the top 500 scoring drug-enzyme interactions.

Subsequently, a comprehensive network describing the drug-target interactions was constructed. In order to make it clear and simple, the top 500 scoring drug-target interactions were used to generate a bipartite graph of drug-target interactions for illustrating the complex relationships between drugs and enzymes, in which a compound and a protein are connected to each other if the protein is a predicted target of the compound. Figure 7 shows a global view of this network with color and shape-coded nodes. To explore the topological and global properties of this drug-target network, the centralization, heterogeneity and node degree distribution were analyzed [44]. The centralization and heterogeneity analysis shows the network centralization and heterogeneity degrees are 0.463 and 3.661, respectively, indicating that a few nodes are more central that the other ones in this net, i.e., the drug-target space is biased toward certain compounds and proteins. Consistent with this, the node degree distribution analysis demonstrates that most nodes have low degrees with only a small proportion of components (hub) interacting with the multiple partners (Figure 8) in this network, and this further suggests that the network is not generated at random. Therefore, we concluded that this predicted drug-target interaction network tends to be controlled by only a small number of drugs and targets, which have a lot of available pharmacological interaction information in the learning dataset.

### Novel Target Prediction for Existing Drugs

Finding new therapeutic indications for the existing drugs represents an efficient parallel approach to the drug discovery, since existing drugs already have extensive clinical history and toxicological information [45]. All above models and the derived information show that new potential drug-target interaction can be effectively predicted by our proposed approach. And to achieve this goal, i.e., to further predict the novel targets for the existing drugs by using our models, two representative small molecules MDMA (DB01454) and Resveratrol (DB02709) were selected presently to illustrate the models' applications, since the comprehensive drug-target interaction network is immensely huge. MDMA is a known psychoactive drug, which is also effective in the treatment of post-traumatic stress disorder [46]. And Resveratrol has the potential of creating anti-inflammatory and anticancer effects [47], [48]. The selection of these two molecules is owing to that their related target information has been reported in literature, but has not been included in training set of the obtained RF Model I and RF Model II. The potential targets of these two molecules are predicted from the pool of all 3987 target proteins using the RF Model I and RF Model II, respectively (with the whole prediction results provided in Supporting Information S11 and S12).

**Figure 9:**
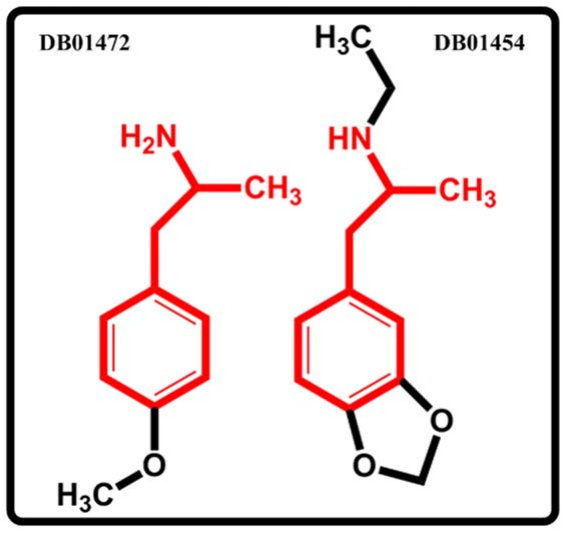
The chemical structures of 4-methoxyamphetamine (DB01472) and MDMA (DB01454).

The obtained results show that MDMA targets 367 different proteins including seven proteins with the binding scores >0.65, i.e., 5-hydroxytryptamine 2A receptor, 5-hydroxytryptamine 2B receptor, 5-hydroxytryptamine 2C receptor, Synaptic vesicular amine transporter, Sodium-dependent serotonin transporter, Sodium- dependent dopamine transporter and Sodium-dependent noradrenaline transporter, all of which receptors have been in fact well demonstrated as the MDMA targets [49]–[53]. Table 4 lists the top 20 scoring targets with the binding scores >0.75. Interestingly, it is found that MDMA binds to the A-1A adrenergic receptor (ADRA1A, binding score = 0.764) and A-2A adrenergic receptor (ADRA2A, binding score = 0.802), which are the targets of 4-methoxyamphetamine (DB01472) [22], a compound structurally similar to MDMA (Figure 9). In addition, this is also consistent with the observation that the MDMA-induced hyperthermia was caused by the activation of ADRA1A, together with the Beta-3 adrenergic receptor [54], [55]. And the Beta-3 adrenergic receptor is also correctly predicted to interact with the MDMA (binding score 0.676). All these suggest the biological relevance of this drug-target interaction prediction.

**Table 4:** Predicted top 20 scoring target proteins of MDMA.

Protein name	UniProt ID	Binding score
Beta-1 adrenergic receptor	P08588	0.820
Carbonic anhydrase 2	P00918	0.810
Prothrombin	P00734	0.804
Alpha-2A adrenergic receptor	P08913	0.802
Prostaglandin G/H synthase 2	P35354	0.800
Dipeptidyl peptidase 4	P27284	0.798
Acetylcholinesterase	P22303	0.796
Nitric-oxide synthase, endothelial	P29474	0.794
Beta-2 adrenergic receptor	P07550	0.794
Prostaglandin G/H synthase 1	P23219	0.784
Gag-Pol polyprotein	P12497	0.778
Nitric oxide synthase, inducible	P35228	0.770
Glutamate receptor 2	P42262	0.770
Alpha-2C adrenergic receptor	P18825	0.768
5-hydroxytryptamine 2A receptor	P28223	0.766
Alpha-1A adrenergic receptor	P35348	0.764
cAMP-dependent protein kinase catalytic subunit alpha	P17612	0.760
Gag-Pol polyprotein	P03366	0.758
Gamma-aminobutyric-acid receptor subunit alpha-1	P14867	0.754
Gag-Pol polyprotein	P03367	0.752

Resveratrol is predicted to interact with 318 different proteins, among which four have the binding score >0.57, i.e., Prostaglandin G/H synthase 1, Prostaglandin G/H synthase 2, Ribosyldihydronicotinamide dehydrogenase [quinone] and Casein kinase II subunit α, and all these proteins have been well demonstrated interacting with this compound [56], [57]. Besides, the proteins of Estrogen receptor, Estrogen receptor beta and Xanthine dehydrogenase/oxidase, which are new to RF Model II, are also predicted to interact with this compound (binding score >0.55). Actually, these predicted interactions have been identified and were annotated in Herbal Ingredients' Targets Database (HITD) (http://lifecenter.sgst.cn/hit/welcome.html), although they have not been collected in the DrugBank database. Interestingly Estrogen receptor and Estrogen receptor beta are also the well-known targets of Diethylstilbestrol (DB00255), a chemical sharing high structure similarity with Resveratrol, which suggests that our prediction methods using the protein-ligand space allows sharing information between unrelated (low sequence similarity) proteins via the similarities shared by their ligands. Additionally, the sequence similarities of Estrogen receptor and Xanthine dehydrogenase/oxidase are only ∼7%; further indicating our approach could identify unrelated target proteins of chemical compounds that the standard similarity-based methods fail to detect. All these outcomes demonstrate that the proposed models could be effective tools to directly explore novel targets for those “old” drugs.

### Novel Drug Prediction for Existing Targets

Most drugs are designed to target a particular protein of certain disease. However, a key feature in the drug-target interaction is that drugs often bind to multiple targets, known as polypharmacology or drug promiscuity. Therefore, the new potential functions of the “old” targets for seeking novel treatment channel of relevant diseases might be acquired through the discovery of novel drugs for those existing targets. In this work, our models have exhibited a powerful ability to predict novel drugs for the “old” targets. Presently, a typical protein of Thymidine kinase from herpes simplex virus (UniProt ID Q9QNF7) [58], which is new to the RF Model III, is taken as an example to clarify the application of our model, since the drug-target network is too huge to be provided completely. The selection of this protein is due to that its related drug information has been reported in literature, but has not been adopted in training of the RF Model III. Using the model, the potential drugs of this protein are predicted from the pool of all 6511 drugs (with all results depicted in Supporting Information S13).

The obtained outcomes demonstrate that this Thymidine kinase is predicted to interact with 1484 different small molecules. By investigating the chemical structure of these compounds, we find that these predicted ligands are structurally diverse but exhibit a non-random and clustering tendency in the structural and physicochemical properties. To further quantitatively describe these properties, a clustering analysis based on the hierarchical cluster algorithm [59], [60] was conducted (The whole clustering analysis information are provided in Supporting Informaton S14, S15, S16). The obtained results show that the compounds are distinctly separated into four clusters, i.e., Cluster A (318 molecules), Cluster B (1129 molecules), Cluster C (24 molecules) and Cluster D (13 molecules) (the detailed clustering analysis results are provided in Supporting Information S17).

**Figure 10:**
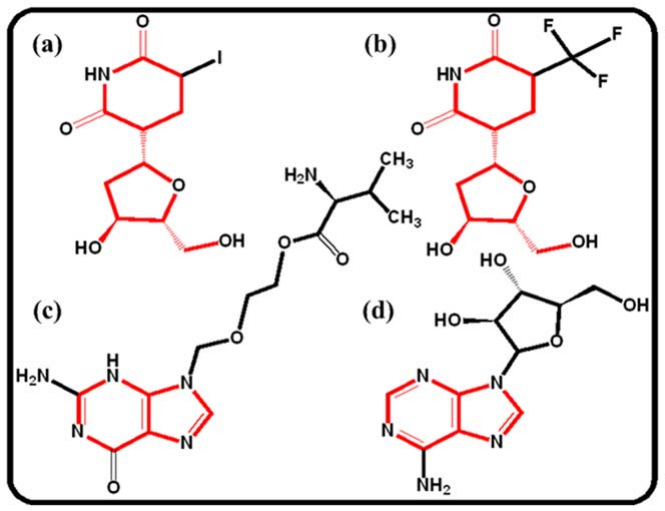
The chemical structure of compounds for (a) Idoxuridine (DB00249), (b) Trifluridine (DB00432), (c) Valaciclovir (DB00577) and (d) Vidarabine (DB00194).

Table 5 shows that the top 10 highest scoring predictions chosen from different ligand families. For cluster A, the structurally similar chemicals NADH (DB00157), Adenosine-5′-Diphosphate (DB03431), Guanosine-5′-Diphosphate (DB03431) and Nicotinamide-Adenine-Dinucleotide (DB01907) are predicted to bind to this enzyme with the binding score >0.80. Actually, Adenosine-5′-Diphosphate is the well-known ligand of a homologous Thymidine kinase (UniProt ID P03176) [22]. With regard to cluster B, compound Idoxuridine (DB00249) is predicted to bind to this enzyme with a binding score 0.762, such interaction has been well-known confirmed and was annotated in DrugBank database [61]. As for cluster C, we do not found any annotated information but it does not mean that they (Hesoheme and Heme) are not potential ligands for this enzyme. In terms of cluster D, this enzyme is found to interact with Pentostatin (DB00552) (binding score 0.656), a chemical sharing a common substructure with Penciclovir (DB00299), which is the known ligand of another homologous Thymidine kinase (UniProt ID P06478) [23]. All these imply that our prediction approaches using the protein-ligand space allows sharing information between scaffold hopping chemicals via the similarities shared by their receptors. Additionally, the other three well-known ligands of this enzyme, i.e., Vidarabine (DB00194), Valaciclovir (DB00577) and Trifluridine (DB00432) [62]–[64], were also screened out with a binding score >0.65. However, as shown in Figure 10, Trifluridine and Idoxuridine are not structurally similar with Vidarabine and Valaciclovir, indicating that our model is useful to identify novel scaffold ligands of the receptors. Besides, it also suggests that the underlying patterns in the multiple drug-target interactions can be captured by this proposed approach. Therefore, we concluded that our approach is capable of detecting novel candidates for those “old” targets.

**Table 5:** Predicted top 10 scoring drugs of Thymidine kinase.

Drug generic name	DrugBank ID	Binding score	Cluster
NADH	DB00157	0.870	A
Nicotinamide-Adenine-Dinucleotide	DB01907	0.848	A
Adenosine-5′-Diphosphate	DB03431	0.844	A
Guanosine-5′-Diphosphate	DB04315	0.808	A
Acetate Ion	DB04184	0.792	B
Mesoheme	DB02577	0.786	C
Heme	DB03014	0.786	C
Idoxuridine	DB00249	0.762	B
Pentostatin	DB00552	0.656	D
1-Beta-Ribofuranosyl-1,3-Diazepinone	DB03185	0.622	D

## Discussion

Traditional drug discovery is largely based upon ‘one molecule-one target-one disease’ model, but there is a growing recognition that drugs work by targeting multiple proteins [65], [66]. The biological network and pathways possessing inherent redundancy and robustness imply that regulating a single target might fall short of producing the desired therapeutic effects [67], [68]. Therefore, the development of multiple drug-target interaction prediction models to investigate disease-associated drug-target network will undoubtedly be an enduring trend for future drug discovery.

In this report, by integrating the information from the chemical structure, protein sequence and pharmacological drug-target interaction data, we developed a set of *in silico* models using a large-scale dataset to predict the potential drug-target interactions. All models were evaluated and verified by both internal and external validations. The outcomes demonstrated the strength of our proposed method for predicting drug-target interaction, which indicates that the conserved binding patterns between drugs and targets can be extracted by our approach from the dataset that contains adequate feature vectors for chemical-protein pairs.

Selecting a suitable encoding of the compounds and proteins information is one of the main computational challenges for the prediction of drug-target interactions using *in silico* tools. In our case, we apply DRAGON molecular descriptors and structural and physicochemical properties descriptors to represent ligands and targets, respectively. Our successful predictions indicate that this adopted chemical and proteins encoding can effectively distinguish the drug-target binding pairs from the non-binding pairs. Additionally, the choice of merging protein and ligand descriptors into a single vector describing both partners (i.e. encode the protein-ligand pair in the joint space) was also adopted in this study, which means that the structural similarity between the two different drugs/targets are independently evaluated by the same measure and are then multiplied to give the overall similarity. Although this description prevents from separate tuning of similarity measures in the protein and ligand spaces when using the SVM kernel approach, it is simple and effective to evaluate the similarity of drug-target pairs [69].

In this study, we proposed the RF approach to predict drug-target interactions, which is a new contribution in the field of drug discovery and development. As well known, RF introduces two sources of randomness into the trees: random training set (bootstrap) and random input vectors. Each tree is grown using a bootstrap sample of training data at each node, with best split chosen from random sample of 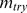 variables instead of all variables. The Bagging (bootstrap aggregating) method causes the low bias and high variance of the unpruned trees, but the variance is reduced by averaging the bootstrapped trees. Because each node is split using the best among a subset of predictors randomly chosen at that node, this strategy turns out to perform very well compared with many other classifiers such as ANN and SVM, and is more robustness against overfitting. However, it should be also pointed out that SVM allows us to use a tensor product space, with no extra calculation time with respect to the joint space, and versatile choice of similarity measures for proteins and ligands, which is a clear advantage of SVM with respect to this proposed RF approach.

As shown in the Results section, the proposed method enables to identify the unrelated proteins that may share structurally similar pockets in the 3D space, the advantage effect of which is derived from the learning ligand similarities. If the unrelated receptors do not possess similar ligands in the learning dataset, then the protein encoding (structural and physicochemical properties derived from their primary sequence) plays a key role for the unrelated targets identification, which have been well applied for the prediction of protein structural and functional classes, protein-protein interactions and subcellular locations [70]–[72]. Particularly, most descriptors of this encoding such as Composition of Solvent Accessibility and Distribution of Charge are highly useful for representing and distinguishing interaction profiles, which is essential for the successful application of statistical learning methods in predicting the interaction profiles between drugs and targets [73]. Although this encoding can effectively describe the interaction profiles of the whole protein, it is limited by the description and extraction of the 3D structure features of the ligand-binding domains. Therefore, obtaining optimal protein encoding method will require further research.

The main advantages of our proposed approach are summarized as follows: 1) In building models, no explicit procedure is needed to select the information shared among all drug-target recognitions. 2) Our system is suitable for simultaneously screening huge numbers of drug candidates and candidate targets from a systematic level; 3) Compared with the structure-based simulation methods, this approach is not limited by the 3D structure data of targets; 4) The structure similarity of a chemical with ligands that bind to proteins which this compound is not known to bind can be used by our method to discover unknown activities. 5) The system is able to identify those ligands/receptors that exhibit scaffold hopping/low sequence similarity; and 6) this approach can aid in discovery of multi-target drugs by recognizing the group of proteins targeted by a particular ligand. Based on all these, we conclude that the present systematic biology-based “integrationist mindset” is appropriate for modeling and understanding complex drug-target interaction networks. This perspective could devote to the recapturing of known small molecules and the explication of mechanisms of drug side effects, and finally is anticipated to help fill in the existing gap between chemical genomics and network pharmacology.

## Supporting Information

Supporting Information S1 The omitted drugs and targets in building models.
(XLSX)

Supporting Information S2 All drug molecules used in building models.
(XLSX)

Supporting Information S3 All target proteins used in building models.
(XLSX)

Supporting Information S4 The omitted drug descriptors in building models.
(XLSX)

Supporting Information S5 All descriptors used in building models.
(XLSX)

Supporting Information S6 Experimental dataset for building models.
(XLSX)

Supporting Information S7 The distribution of all samples of Model II using the first three principle components.
(TIF)

Supporting Information S8 The distribution of all samples of Model III using the first three principle components.
(TIF)

Supporting Information S9 The distribution of all samples of Model IV using the first three principle components.
(TIF)

Supporting Information S10 The blind testing dataset obtained from the KEGG database.
(XLSX)

Supporting Information S11 The predicted targets for MDMA by RF Model I.
(XLSX)

Supporting Information S12 The predicted targets for Resveratrol by RF Model II.
(XLSX)

Supporting Information S13 The predicted drugs for Thymidine kinase by RF Model III.
(XLSX)

Supporting Information S14 The detailed clustering analysis information.
(DOC)

Supporting Information S15 The adopted descriptors along with definition and category information.
(XLSX)

Supporting Information S16 The dendrogram shows the hierarchical clustering of 1484 predicted drugs. Clusters A, B, C and D include 318, 1129, 24 and 13 compounds, respectively.
(TIF)

Supporting Information S17 The clustering analysis results of the predicted drugs for Thymidine kinase.
(XLSX)
