# Supplementary figures and images for: A Systematic Prediction of Multiple Drug-Target Interactions from Chemical, Genomic, and Pharmacological Data
Source: PLoS One. 2012 May 30;7(5):e37608. doi: 10.1371/journal.pone.0037608 (PMC3364341; doi:10.1371/journal.pone.0037608)

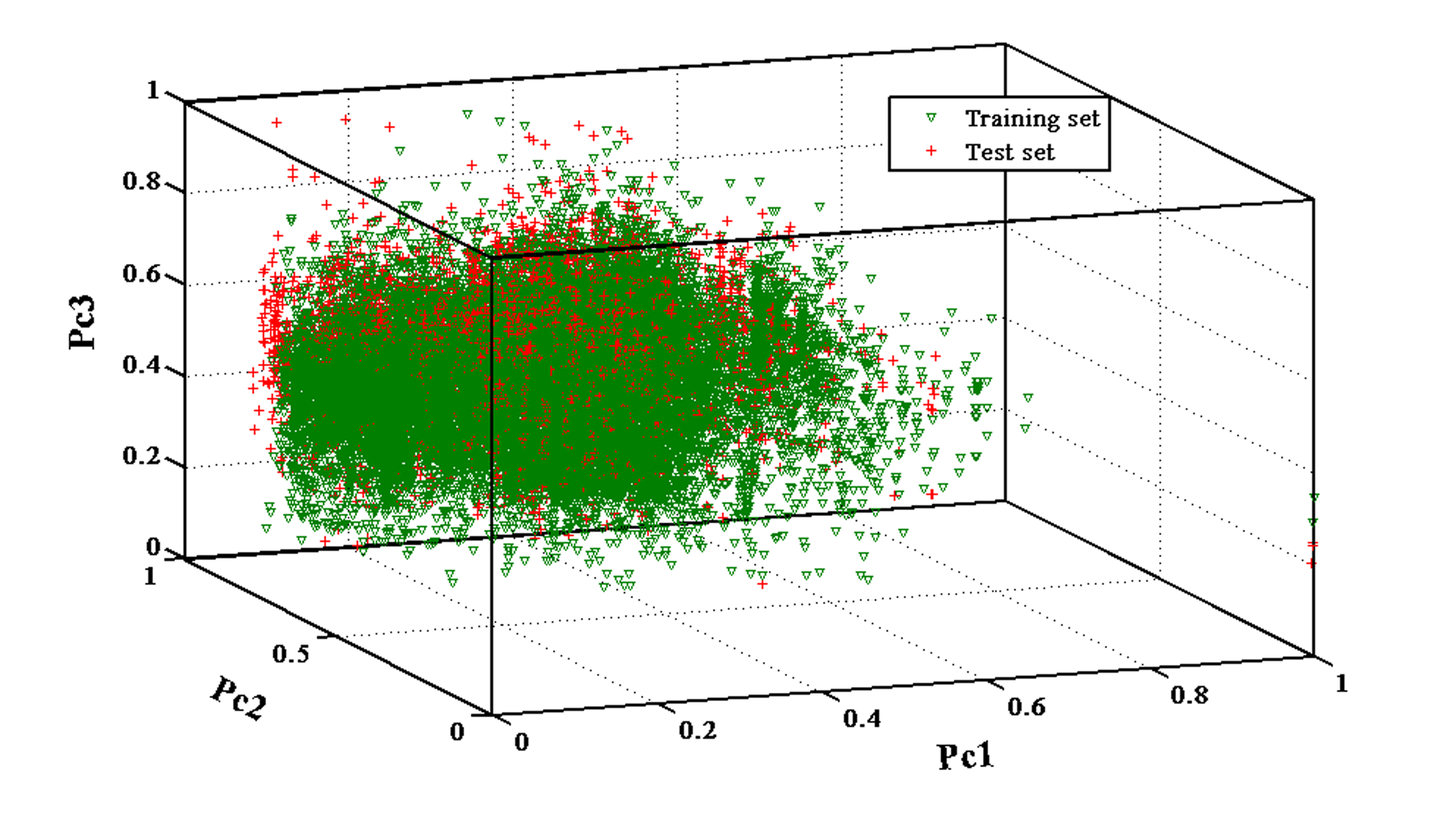

Supplement: Supporting Information S7 — The distribution of all samples of Model II using the first three principle components. (TIF) [file pone.0037608.s007.tif]

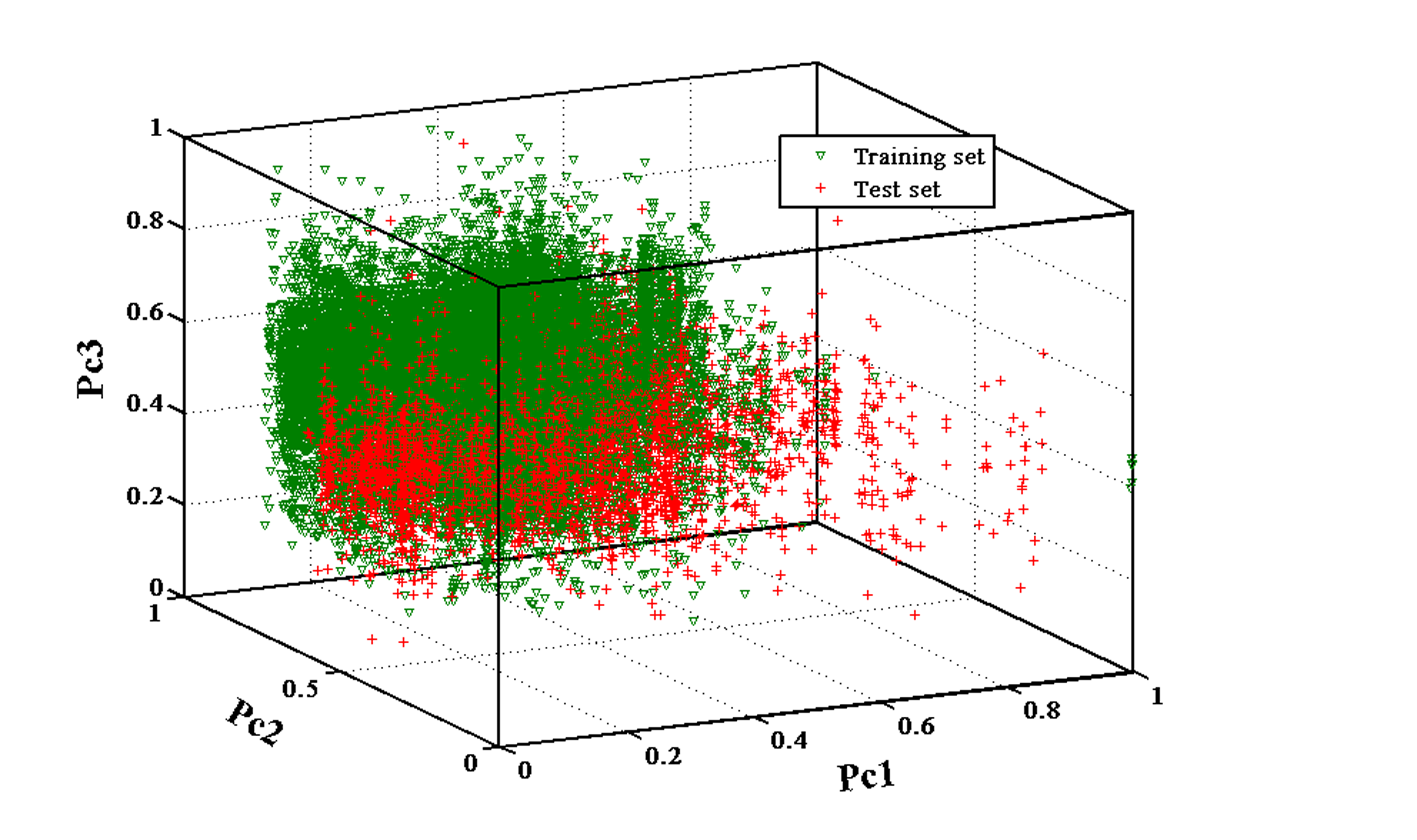

Supplement: Supporting Information S8 — The distribution of all samples of Model III using the first three principle components. (TIF) [file pone.0037608.s008.tif]

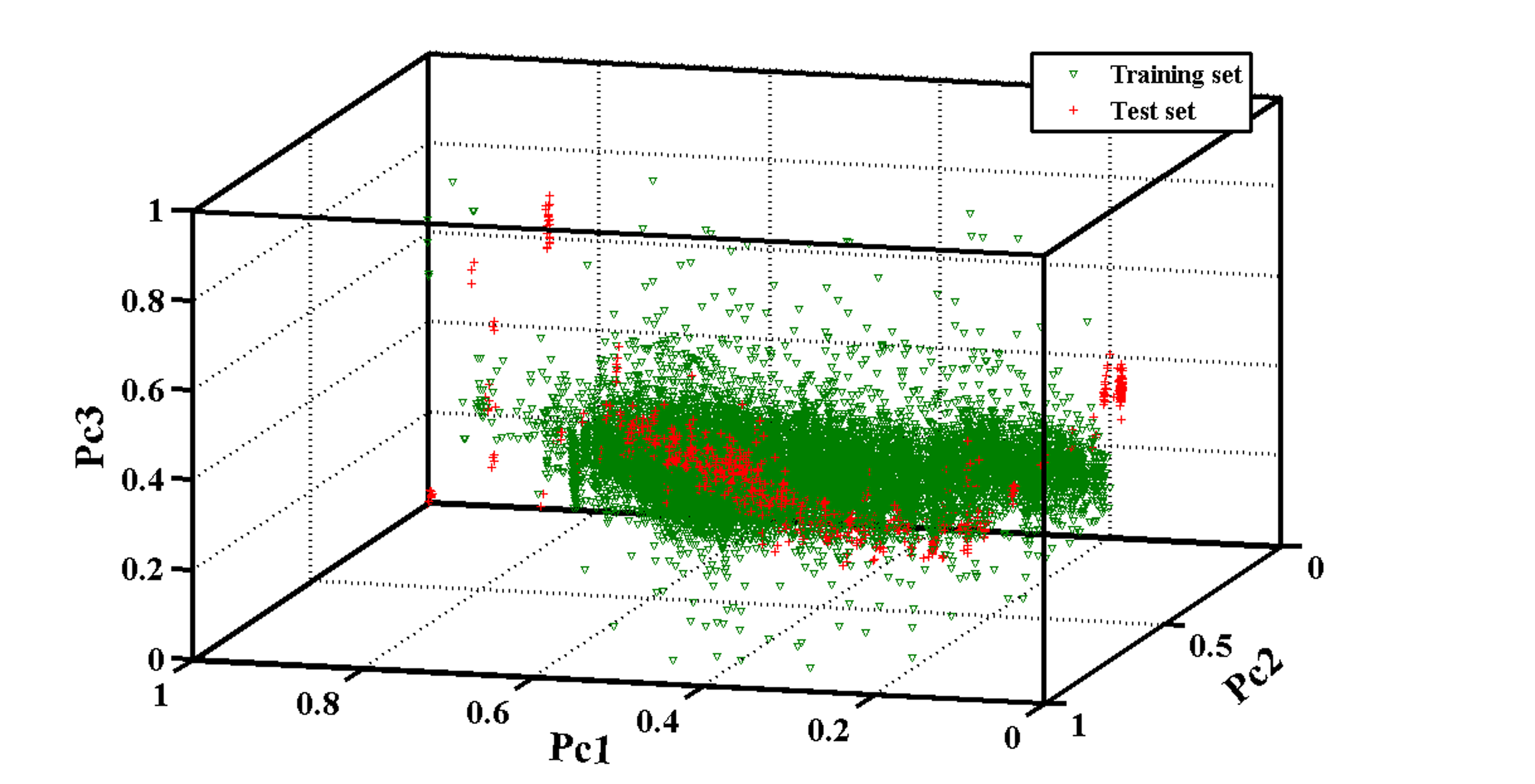

Supplement: Supporting Information S9 — The distribution of all samples of Model IV using the first three principle components. (TIF) [file pone.0037608.s009.tif]

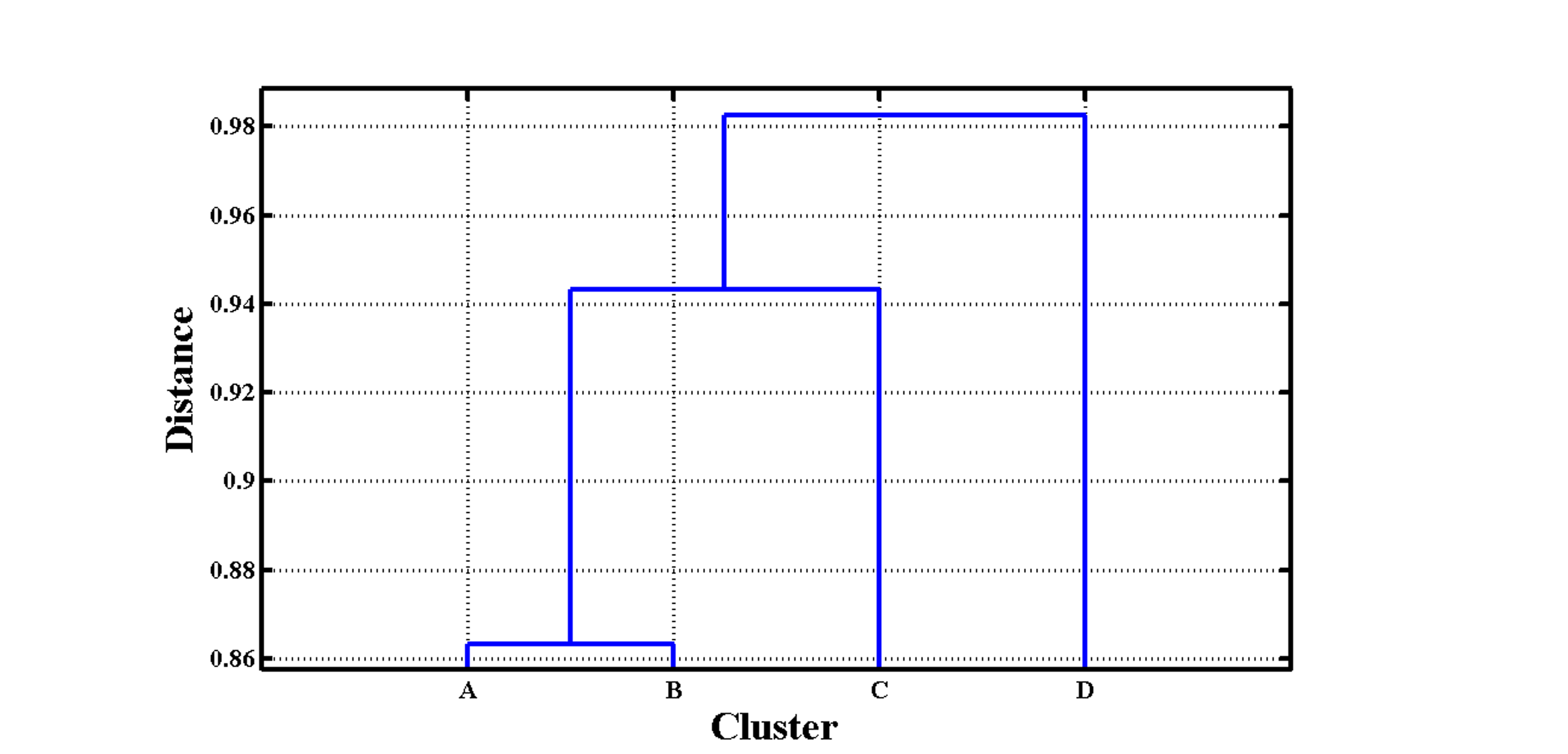

Supplement: Supporting Information S16 — The dendrogram shows the hierarchical clustering of 1484 predicted drugs. Clusters A, B, C and D include 318, 1129, 24 and 13 compounds, respectively. (TIF) [file pone.0037608.s016.tif]
