# Supplementary material for: A Systematic Prediction of Multiple Drug-Target Interactions from Chemical, Genomic, and Pharmacological Data
Source: PLoS One. 2012 May 30;7(5):e37608. doi: 10.1371/journal.pone.0037608 (PMC3364341; doi:10.1371/journal.pone.0037608)
Supplement: Supporting Information S14 — The detailed clustering analysis information. (DOC) [file pone.0037608.s014.doc]

**Clustering Analysis**

To carry out the clustering process, the average linkage method is implemented and the distance metric used is 1-*r* in which *r* is the Pearson correlation coefficient between the molecular descriptors of two compounds. The 20 molecular descriptors (EEig04r, nArC=N, GATS3m, O-062, nR11, Mor20v, G(N..P), GATS2v, R1p, Mor04e, D/Dr11, R4p+, GATS1v, Mor27m, Mor17p, GATS2m, D/Dr07, GATS4e, T(N..P) and Mor18u) were employed. These descriptors are distributed in different categories including the constitutional parameters, topological variables, edge adjacency indices, functional group counts, 2D autocorrelations, 3D-MoRSE descriptors, geometrical descriptors, GETAWAY descriptors and atom-centered fragment (with details referred to Supplementary Material S15).

The obtained results show that the compounds are distinctly separated into four clusters (Supplementary Material S16). Cluster A is comprised of those compounds which contain phosphate groups as indicated by descriptors of G(N..P) (Sum of the geometrical distances between N..P), and T(N..P) (Sum of the topological distances between N..P). Cluster B is characterized by the descriptors of Mor27m (3D-MoRSE-signal 27/weighted by mass), R1p (R autocorrelation of lag 1/weighted by polarizability) and Mor17p (3D-MoRSE-signal 17/weighted by polarizability) with lower values, indicating that these molecules are smaller in size and more non-polar. Cluster C is comprised of compounds with the metal ion contained heterocyclic ring, as depicted by D/Dr07 (Distance/detour ring index of order 7) which is all larger than 33.90. And Cluster D is mainly made up of polycyclics-like compounds, which contain two or more nitrogen-containing heterocyclic rings fused together as reflected by the descriptors D/Dr11 (Distance/detour ring index of order 11) and nR11 (Number of 11-membered rings) This indicates that the predicted ligands of the enzyme cluster into different groups related with specific physicochemical properties. Additionally, novel therapeutic agents may be sought by studying certain representative members of these clusters, since the clustering analysis can effectively extract the connection between the physicochemical properties and activities.
